# Supplementary figures and images for: Potent immunogenicity and protective efficacy of a multi-pathogen vaccination targeting Ebola, Sudan, Marburg and Lassa viruse
Source: PLoS Pathog. 2024 Jun 26;20(6):e1012262. doi: 10.1371/journal.ppat.1012262 (PMC11233014; doi:10.1371/journal.ppat.1012262)

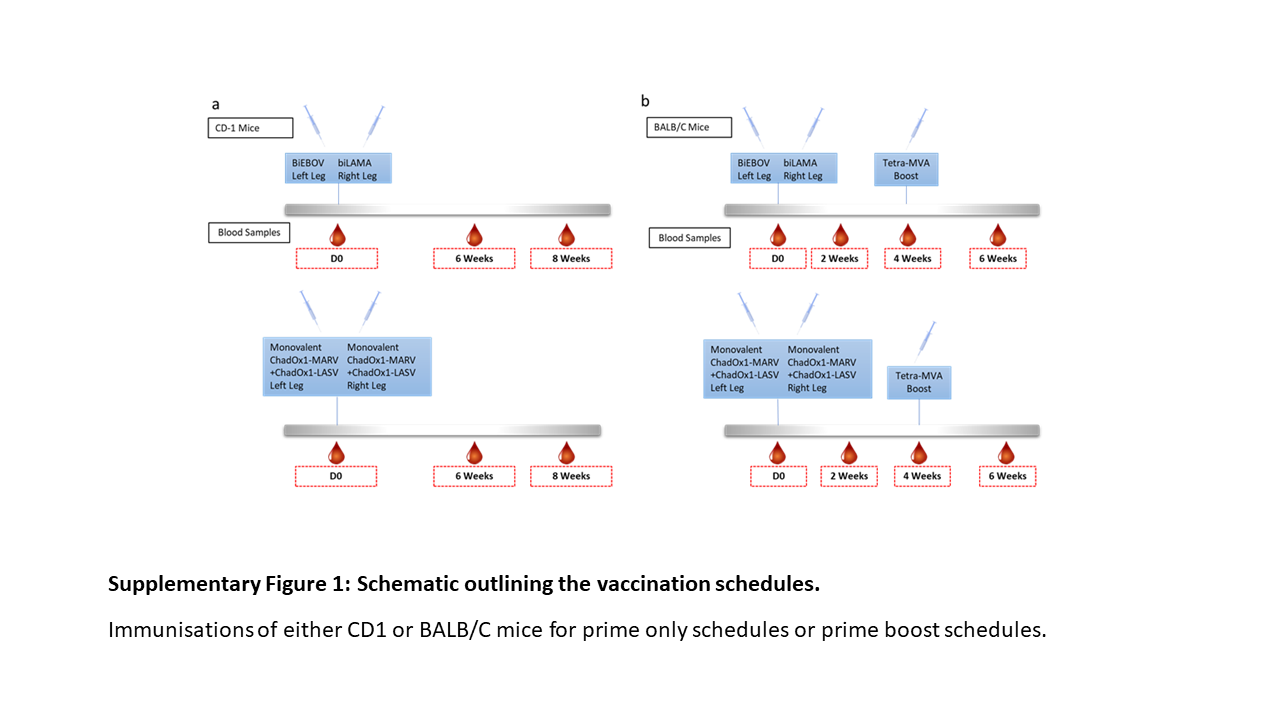

Supplement: S1 Fig — Immunisations of either CD1 or BALB/C mice for prime only schedules or prime boost schedules. (TIF) [file ppat.1012262.s001.TIF]

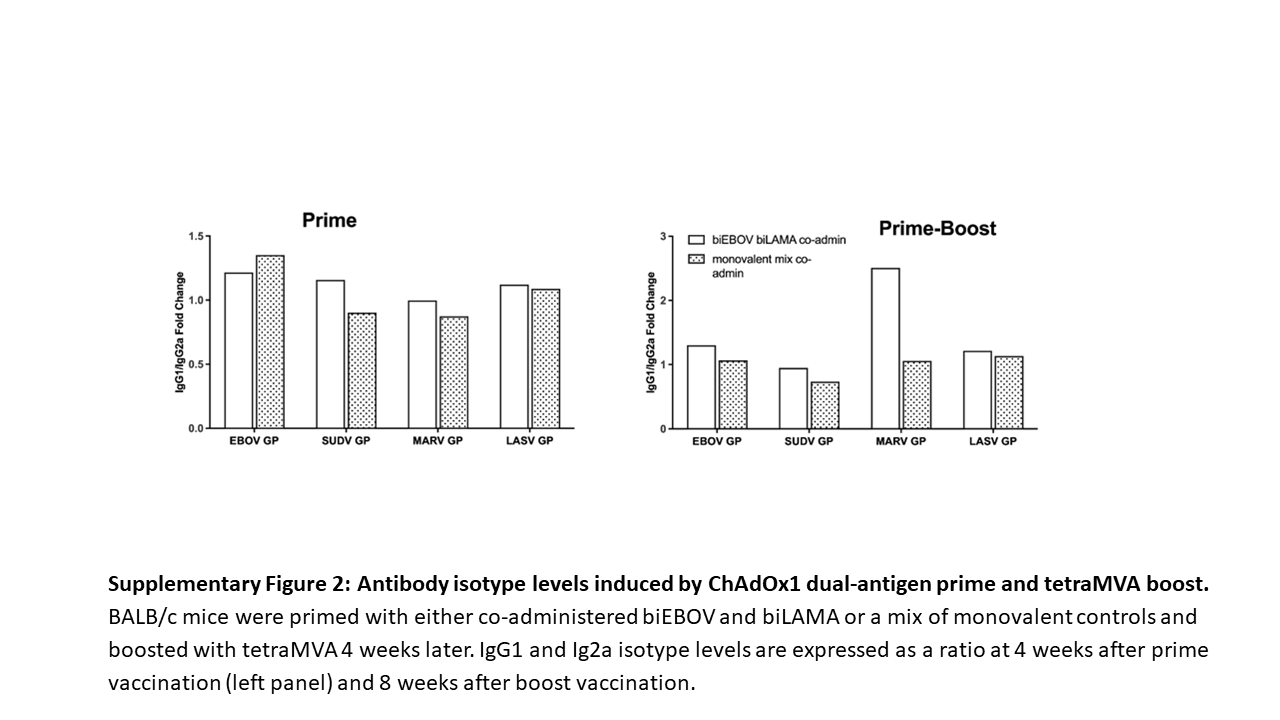

Supplement: S2 Fig — BALB/c mice were primed with either co-administered biEBOV and biLAMA or a mix of monovalent controls and boosted with tetraMVA 4 weeks later. IgG1 and Ig2a isotype levels are expressed as a ratio at 4 weeks after prime vaccination (left panel) and 8 weeks after boost vaccination. (TIF) [file ppat.1012262.s002.TIF]

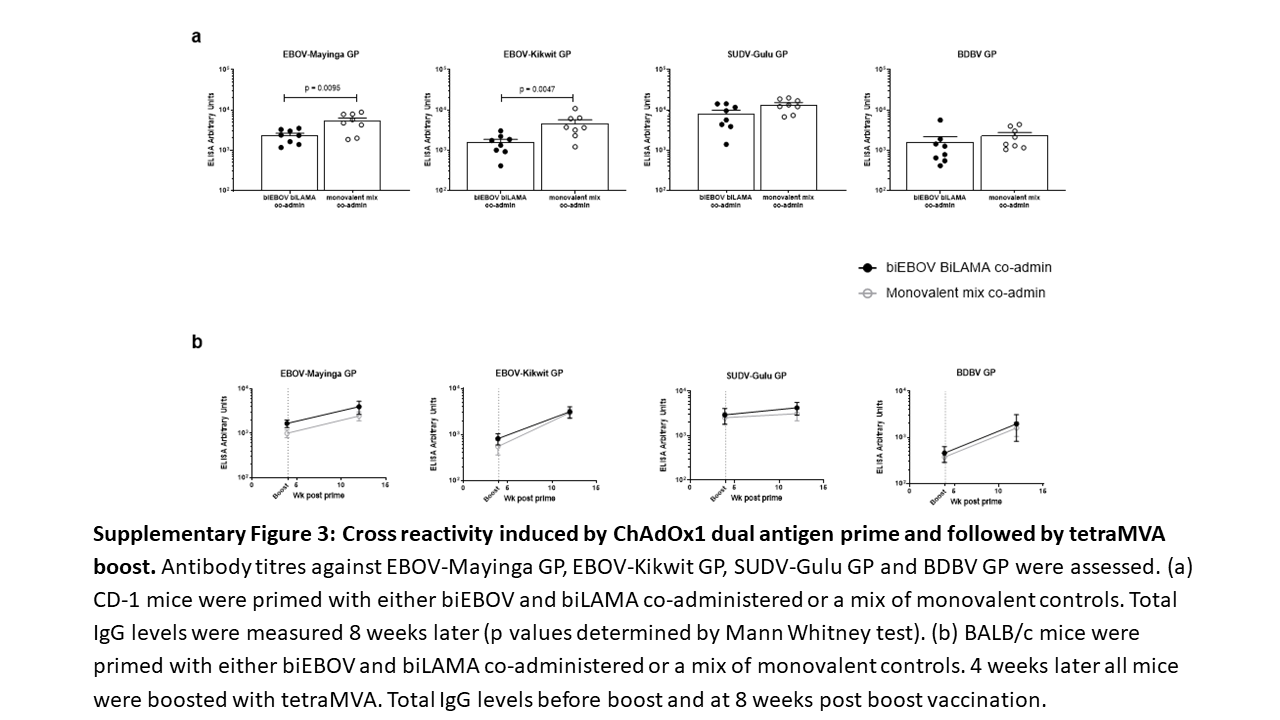

Supplement: S3 Fig — Antibody titres against EBOV-Mayinga GP, EBOV-Kikwit GP, SUDV-Gulu GP and BDBV GP were assessed. (a) CD-1 mice were primed with either biEBOV and biLAMA co-administered or a mix of monovalent controls. Total IgG levels were measured 8 weeks later (p values determined by Mann Whitney test). (b) BALB/c mice were primed with either biEBOV and biLAMA co-administered or a mix of monovalent controls. 4 weeks later all mice were boosted with tetraMVA. Total IgG levels before boost and at 8 weeks post boost vaccination. (TIF) [file ppat.1012262.s003.TIF]

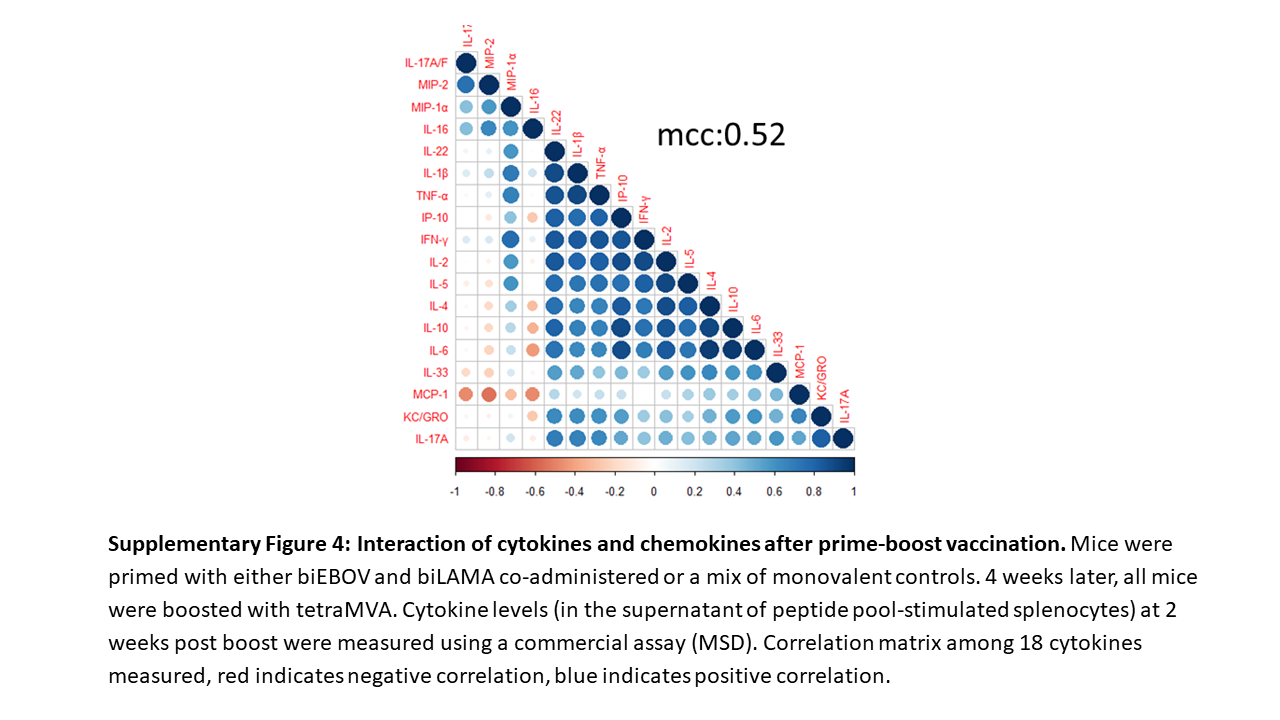

Supplement: S4 Fig — Mice were primed with either biEBOV and biLAMA co-administered or a mix of monovalent controls. 4 weeks later, all mice were boosted with tetraMVA. Cytokine levels (in the supernatant of peptide pool-stimulated splenocytes) at 2 weeks post boost were measured using a commercial assay (MSD). Correlation matrix among 18 cytokines measured, red indicates negative correlation, blue indicates positive correlation. (TIF) [file ppat.1012262.s004.TIF]

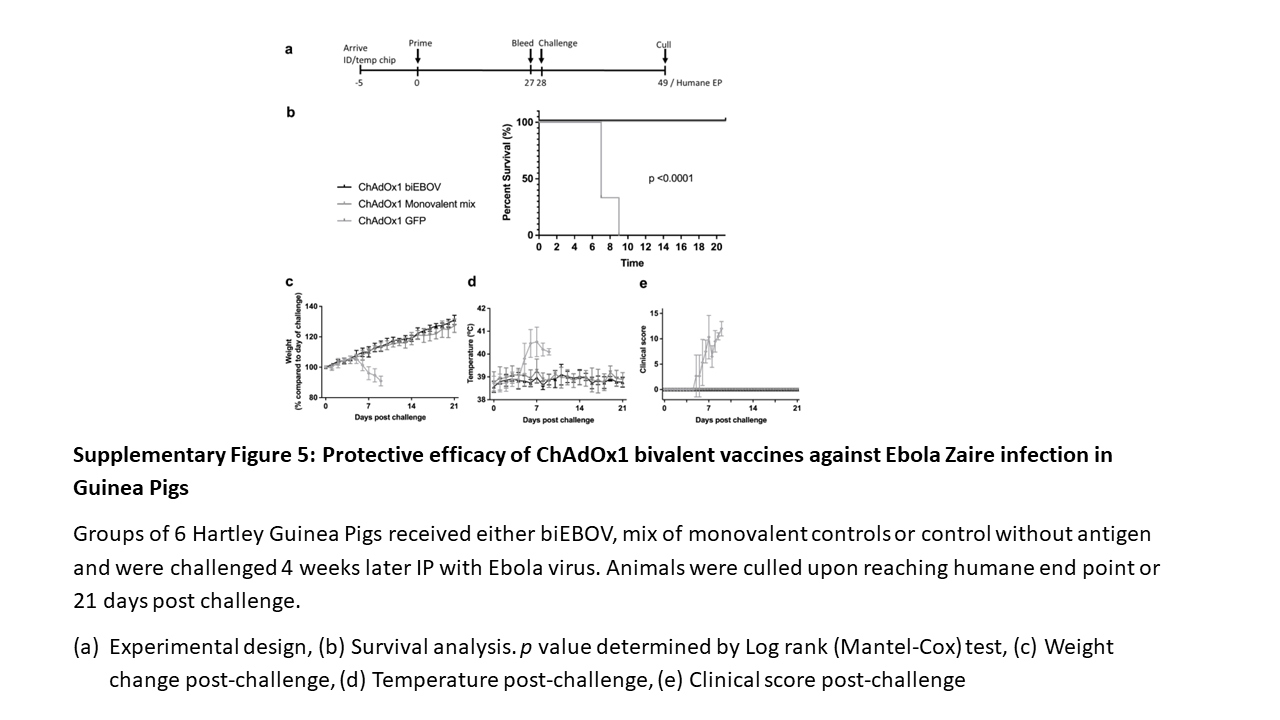

Supplement: S5 Fig — Groups of 6 Hartley Guinea Pigs received either biEBOV, mix of monovalent controls or control without antigen and were challenged 4 weeks later IP with Ebola virus. Animals were culled upon reaching humane end point or 21 days post challenge. (a). Experimental design. (b). Survival analysis. p value determined by Log rank (Mantel-Cox) test. (c). Weight change post challenge. (d). Temperature post challenge. (e). Clinical score post challenge. (TIF) [file ppat.1012262.s005.TIF]

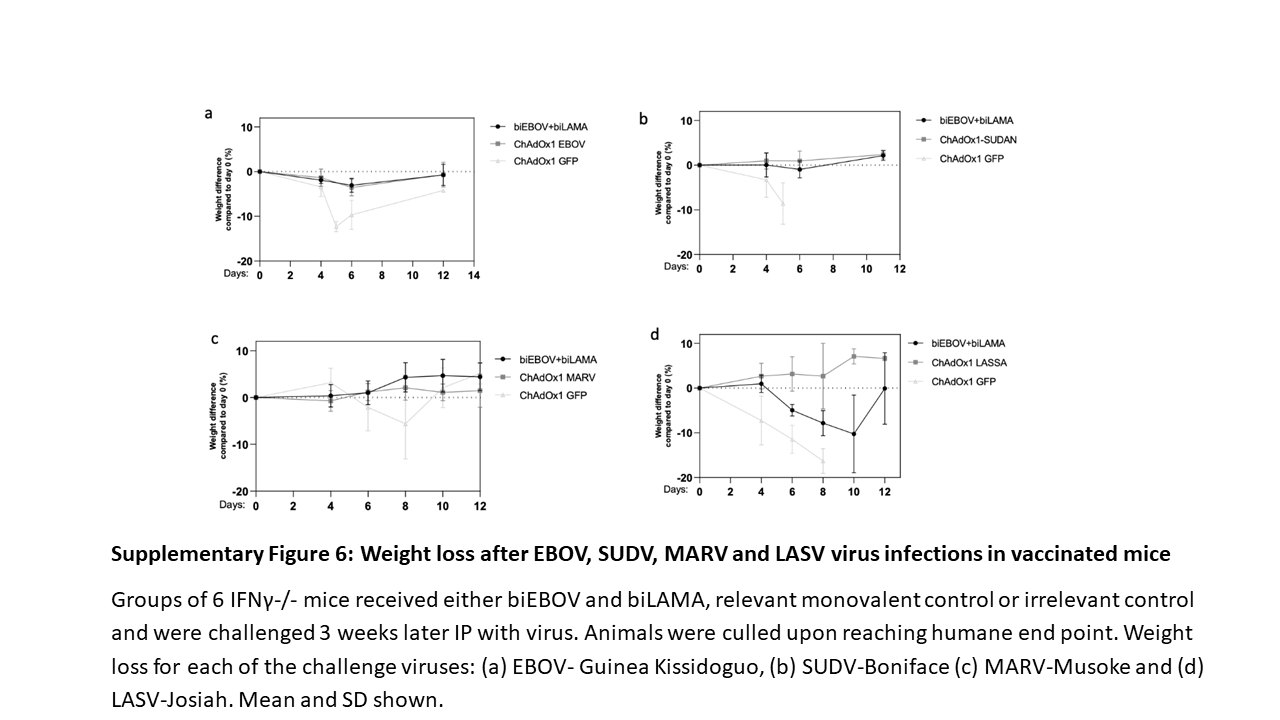

Supplement: S6 Fig — Groups of 6 IFNγ-/- mice received either biEBOV and biLAMA, relevant monovalent control or irrelevant control and were challenged 3 weeks later IP with virus. Animals were culled upon reaching humane end point. Weight loss for each of the challenge viruses: (a) EBOV-Guinea Kissidoguo, (b) SUDV-Boniface (c) MARV-Musoke and (d) LASV-Josiah. Mean and SD shown. (TIF) [file ppat.1012262.s006.TIF]

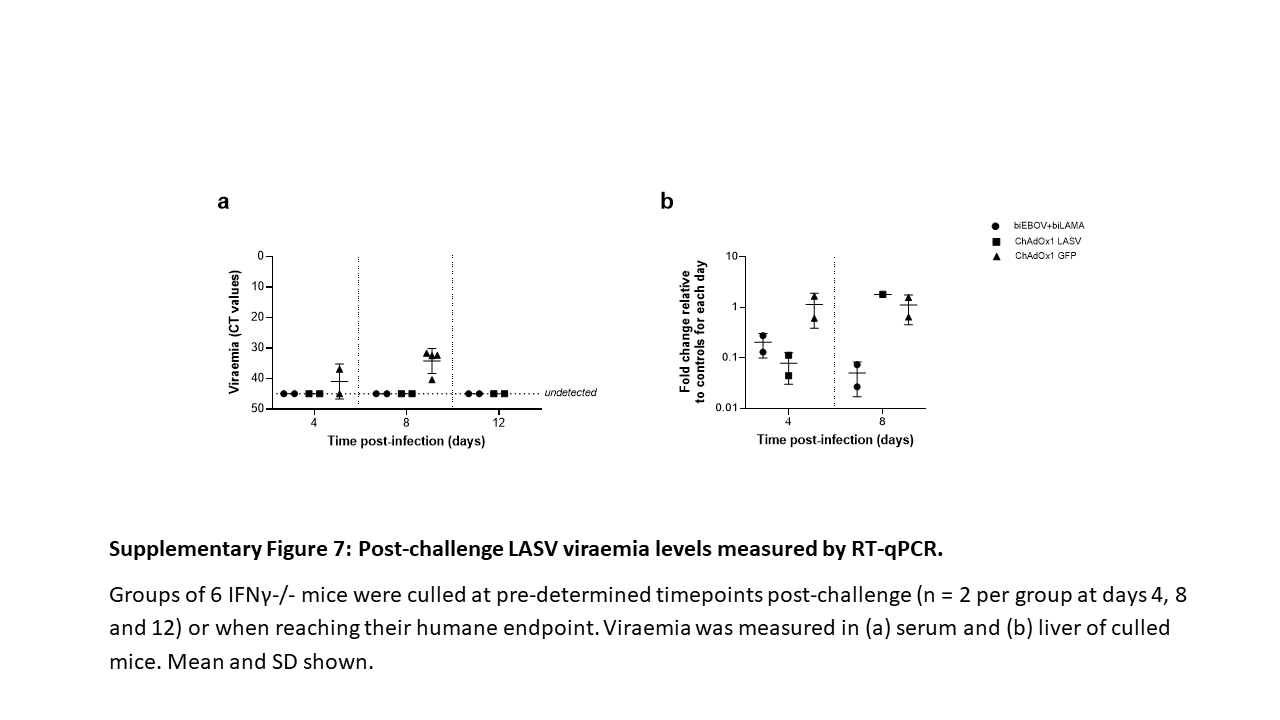

Supplement: S7 Fig — Groups of 6 IFNγ-/- mice were culled at pre-determined timepoints post-challenge (n = 2 per group at days 4, 8 and 12) or when reaching their humane endpoint. Viraemia was measured in (a) serum and (b) liver of culled mice. Mean and SD shown. (TIF) [file ppat.1012262.s007.TIF]

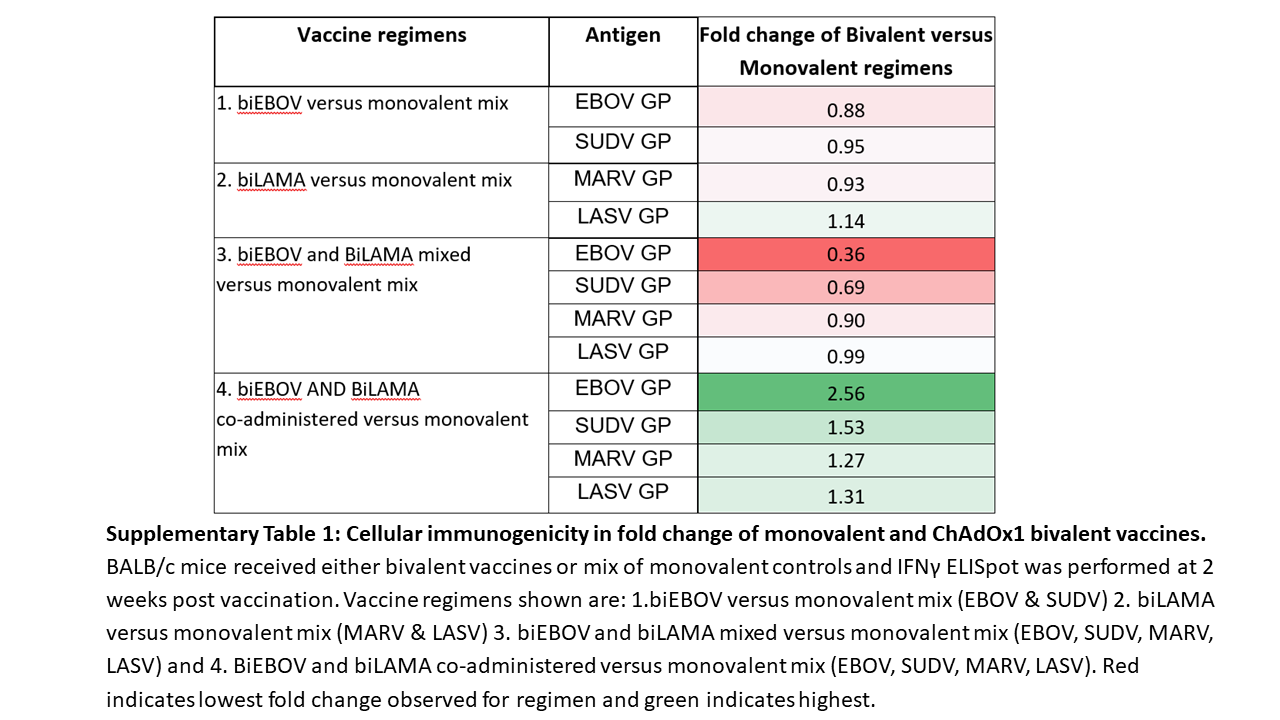

Supplement: S1 Table — BALB/c mice received either bivalent vaccines or mix of monovalent controls and IFNγ ELISpot was performed at 2 weeks post vaccination. Vaccine regimens shown are: 1.biEBOV versus monovalent mix (EBOV & SUDV) 2. biLAMA versus monovalent mix (MARV & LASV) 3. biEBOV and biLAMA mixed versus monovalent mix (EBOV, SUDV, MARV, LASV) and 4. EBOV and biLAMA co-administered versus monovalent mix (EBOV, SUDV, MARV, LASV). Red indicates lowest fold change observed for regimen and green indicates highest. (TIF) [file ppat.1012262.s008.TIF]

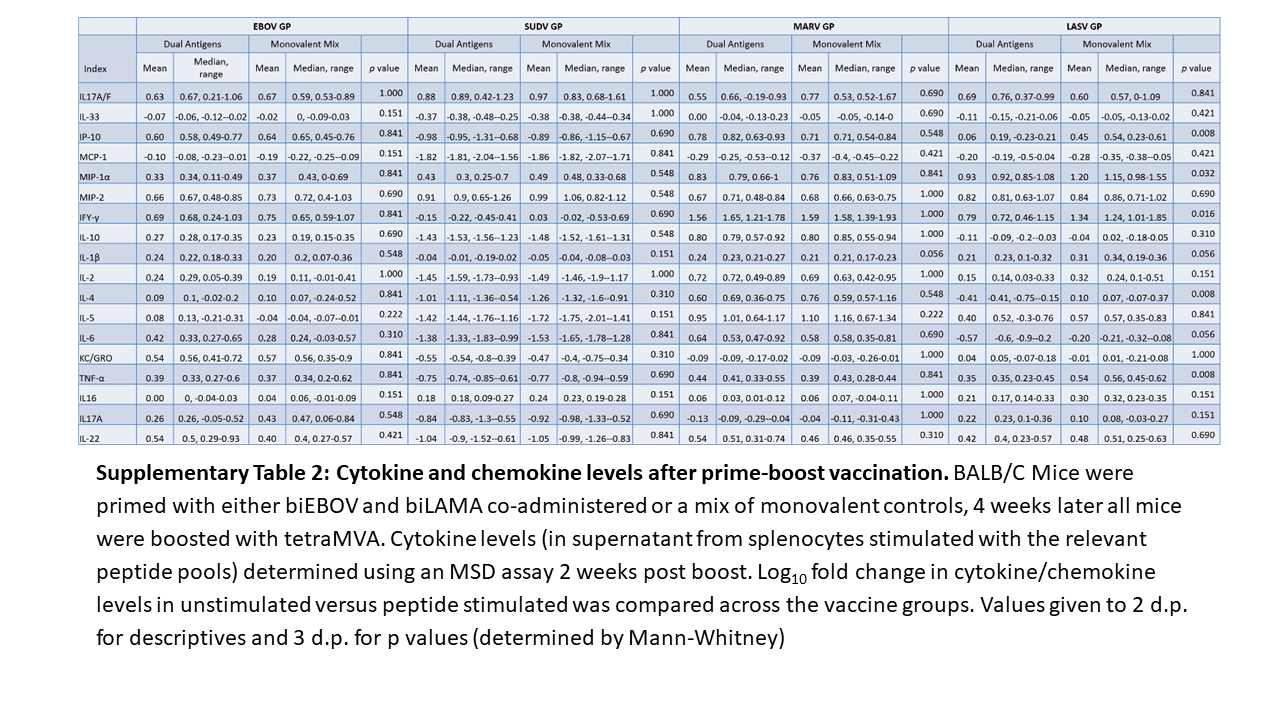

Supplement: S2 Table — BALB/C Mice were primed with either biEBOV and biLAMA co-administered or a mix of monovalent controls, 4 weeks later all mice were boosted with tetraMVA. Cytokine levels (in supernatant from splenocytes stimulated with the relevant peptide pools) determined using an MSD assay 2 weeks post boost. Log10 fold change in cytokine/chemokine levels in unstimulated versus peptide stimulated was compared across the vaccine groups. Values given to 2 d.p. for descriptives and 3 d.p. for p values (determined by Mann-Whitney). (TIF) [file ppat.1012262.s009.TIF]
